# Extracellular vesicles in cardiovascular diseases

**DOI:** 10.1038/s41420-020-00305-y

**Published:** 2020-07-30

**Authors:** Shihui Fu, Yujie Zhang, Yulong Li, Leiming Luo, Yali Zhao, Yao Yao

**Affiliations:** 1 Department of Geriatric Cardiology, Chinese People’s Liberation Army General Hospital, Beijing, 100853 China; 2 Department of Cardiology, Hainan Hospital of Chinese People’s Liberation Army General Hospital, Sanya, 572013 China; 3 Department of Epidemiology, School of Public Health, Southern Medical University, Guangzhou, 510515 China; 4 Central Laboratory, Hainan Hospital of Chinese People’s Liberation Army General Hospital, Sanya, 572013 China; 5 Centre for the Study of Ageing and Human Development and Geriatrics Division, Medical School of Duke University, Durham, NC 27708 USA; 6 Centre for Healthy Ageing and Development Studies, National School of Development, Peking University, Beijing, 100871 China

**Keywords:** Molecular biology, Cardiovascular diseases

## Abstract

Due to the continued high incidence and mortality rate worldwide, there is still a need to develop new strategies for the prevention, diagnosis and treatment of cardiovascular diseases (CVDs). Proper cardiovascular function depends on the coordinated interplay and communication between cardiomyocytes and noncardiomyocytes. Extracellular vesicles (EVs) are enclosed in a lipid bilayer and represent a significant mechanism for intracellular communication. By containing and transporting various bioactive molecules, such as micro-ribonucleic acids (miRs) and proteins, to target cells, EVs impart favourable, neutral or detrimental effects on recipient cells, such as modulating gene expression, influencing cell phenotype, affecting molecular pathways and mediating biological behaviours. EVs can be released by cardiovascular system-related cells, such as cardiomyocytes, endotheliocytes, fibroblasts, platelets, smooth muscle cells, leucocytes, monocytes and macrophages. EVs containing miRs and proteins regulate a multitude of diverse functions in target cells, maintaining cardiovascular balance and health or inducing pathological changes in CVDs. On the one hand, miRs and proteins transferred by EVs play biological roles in maintaining normal cardiac structure and function under physiological conditions. On the other hand, EVs change the composition of their miR and protein cargoes under pathological conditions, which gives rise to the development of CVDs. Therefore, EVs hold tremendous potential to prevent, diagnose and treat CVDs. The current article reviews the specific functions of EVs in different CVDs.

## FACTS

- By containing and transporting various bioactive molecules, extracellular vesicles represent a significant mechanism for intracellular communication and impart favourable, neutral or detrimental effects on recipient cells, such as modulating gene expression, influencing cell phenotype, affecting molecular pathways and mediating biological behaviours.
- Extracellular vesicles play significant roles in maintaining normal cardiac structure and function under physiological conditions and change their composition under pathological conditions to promote the development of cardiovascular diseases.
- Cardiovascular diseases are the leading cause of death, accounting for almost a third of deaths worldwide. Extracellular vesicles play fundamental roles in regulating physiological and pathological functions and hold tremendous potential to monitor and treat cardiovascular diseases.

## Open questions

- There is still a need to develop new strategies for the prevention and treatment of different cardiovascular diseases, given their continued high incidence and mortality rate worldwide.
- Little is known about extracellular vesicle-mediated regulation of cardiomyocytes and noncardiomyocytes within the healthy and diseased heart.
- There is a lack of broad and detailed understanding of extracellular vesicle functions and a need for the development of new and personalized extracellular vesicle-based therapies to treat different cardiovascular diseases.

## Introduction

**Fig. 1:**
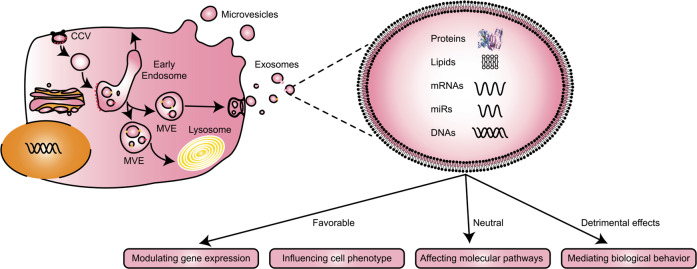
Origins, contents and roles of extracellular vesicles. Extracellular vesicles (EVs) are enclosed in a lipid bilayer and represent a by-product released by cells. In addition to direct cell-cell contact or the transport of secreted molecules, EVs also participate in intercellular communication. By containing and transporting various bioactive molecules, such as proteins, lipids, messenger ribonucleic acids (mRNAs), micro-ribonucleic acids (microRNAs, miRs) and deoxyribonucleic acids (DNAs), to target cells, EVs impart favourable, neutral or detrimental effects on recipient cells, such as modulating gene expression, influencing cell phenotype, affecting molecular pathways and mediating biological behaviours.

Cardiovascular diseases (CVDs) are the leading cause of death, accounting for almost a third of deaths worldwide^1^. Although advances in cardiovascular research and care have increased patient survival, there is still a need to develop new strategies for CVD prevention and treatment, given the continued high incidence and mortality rate in many countries^2^. Extracellular vesicles (EVs) are enclosed in a lipid bilayer and represent a by-product released by cells (Fig. 1)^3^. In addition to direct cell–cell contact or the transport of secreted molecules, EVs also participate in intercellular communication^4^. By containing and transporting various bioactive molecules, such as proteins, lipids, messenger ribonucleic acids (mRNAs), micro-ribonucleic acids (microRNAs, miRs) and deoxyribonucleic acids (DNAs), to target cells, EVs impart favourable, neutral or detrimental effects on recipient cells, such as modulating gene expression, influencing cell phenotype, affecting molecular pathways and mediating biological behaviours^5^.

EVs encompass three subtypes, exosomes, microvesicles and apoptotic bodies, which have different routes of intracellular formation, sizes and contents^6^. Whereas exosomes are released continuously from cells, microvesicles and apoptotic bodies are predominantly released from activated or apoptotic cells. Exosomes are released from cells via the endolysosomal pathway, and microvesicles and apoptotic bodies are formed by budding from the plasma membrane. Apoptotic bodies (>1000 nm) form by apoptotic cell membrane blebbing to recycle contents and contain cell debris, genomic DNA (gDNA) and proteins. Microvesicles (150–1000 nm) form through outward pinching off of the plasma membrane and contain RNAs, proteins and lipids. Exosomes, the smallest type of EV (30–150 nm), originate within multivesicular bodies and carry RNAs, proteins and lipids. The plasma membrane buds inward, fills with cytoplasmic contents and retains plasma membrane proteins specific to the cell of origin. Early endosomes are formed and then mature into late endosomes. This plasma membrane further buds inward and forms intraluminal vesicles with the aid of endosomal sorting complex required for transport. Once filled with these exosomes, late endosomes become multivesicular bodies, which might either fuse with the plasma membrane and secrete exosomes or deliver their contents to the lysosome for degradation. Molecular mechanisms controlling exosome production are far from fully understood, and some of these mechanisms appear to vary between cell types.

**Fig. 2:**
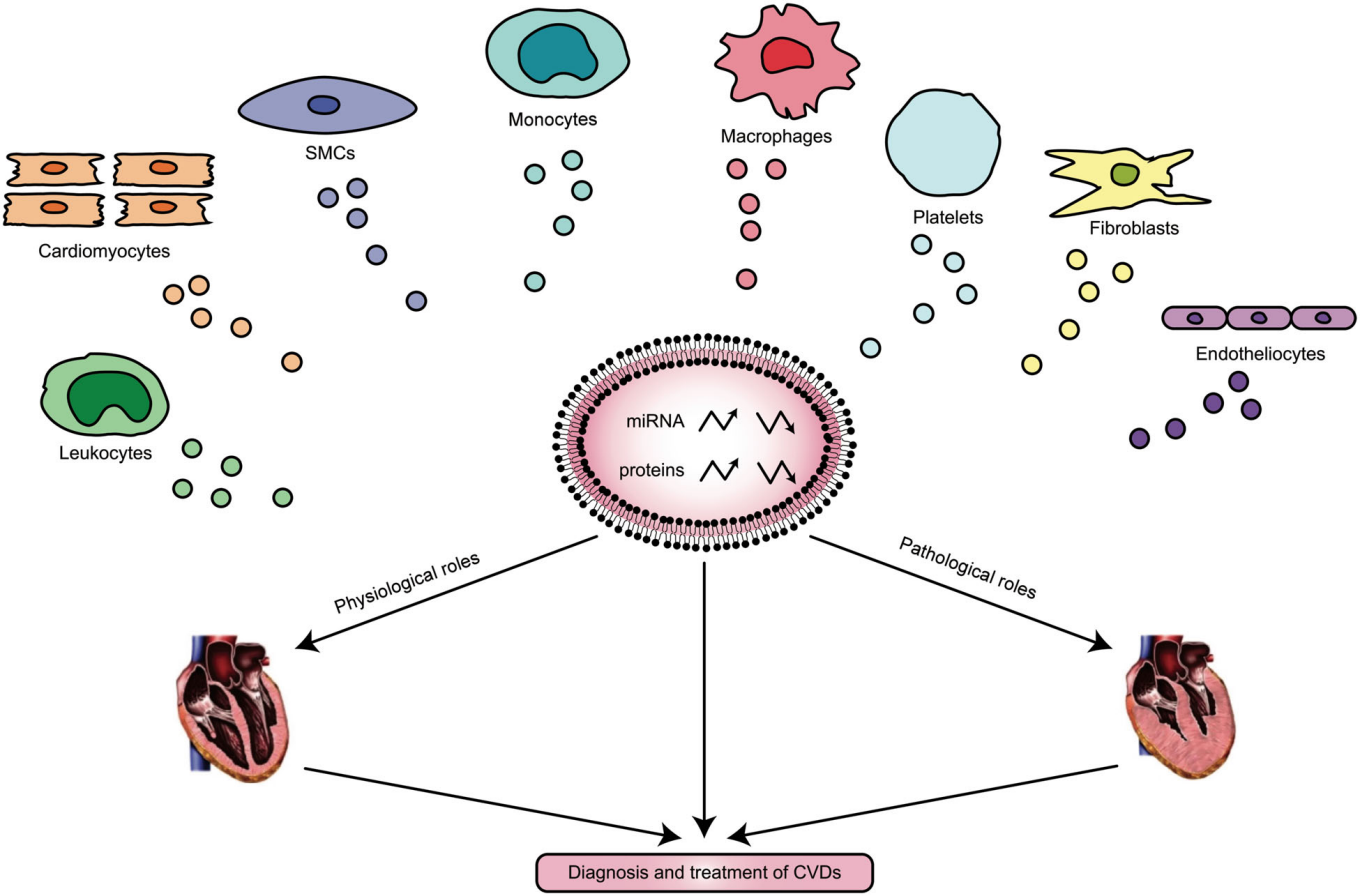
Origins, contents and roles of extracellular vesicles in cardiovascular diseases. Extracellular vesicles (EVs) can be released by cardiovascular system-related cells, such as cardiomyocytes, endotheliocytes, fibroblasts, platelets, smooth muscle cells (SMCs), leukocytes, monocytes and macrophages. On the one hand, EVs play biological roles in maintaining normal cardiac structure and function under physiological conditions. On the other hand, EVs change their composition under pathological conditions and contribute to the development of cardiovascular diseases (CVDs). Therefore, EVs hold tremendous potential to monitor and treat CVDs.

Several studies have recently indicated that EVs can be released by cardiovascular system-related cells, such as cardiomyocytes, endotheliocytes, fibroblasts, platelets, smooth muscle cells (SMCs), leucocytes, monocytes and macrophages (Fig. 2)^7^. On the one hand, EVs play biological roles in maintaining normal cardiac structure and function under physiological conditions. On the other hand, EVs change their composition under pathological conditions and contribute to the development of CVDs^8^. Therefore, EVs hold tremendous potential to monitor and treat CVDs. Increasing evidence suggests that the effects of EVs on target cells are mainly dependent on miRs and proteins transferred by EVs^9^. Depending on the condition of the source cells, EVs containing miRs and proteins have been shown to regulate a multitude of diverse functions in target cells, maintaining cardiovascular balance and health or inducing pathological changes in CVDs (Table 1). The current article reviews the specific functions of EVs in different CVDs^10^.

**Table 1:** Extracellular vesicle-based miRs and targets in cardiovascular diseases.

Cardiovascular diseases	MiRs	Targets
Myocardial ischaemia and injury	MiR-92a-3p^12^; miR-939^13^; miR-126^17^; miR-199a^19^; miR-222^20^; miR-503^21^; miR-143^22^ and miR-125b^24^	Mitochondrially encoded cytochrome c oxidase I^15^; stromal cell-derived factor-1^18^; intercellular adhesion molecule 1^20^; ephrin-B2^21^; vascular endothelial growth factor^21^ and Kruppel-like factor 2^22^
Atrial fibrillation	MiR-199a-3p^29^; miR-21-3p^31^	Gata-binding 4^29^
Cardiac hypertrophyand failure	MiR-21-5p^30^; miR-21-3p^31^; miR-320^33^ and miR-146a^34^	Sorbin and SH3 domain containing protein 2^31^; signal transducer and activator of transcription 3^32^ and small ubiquitin-like proteins modifier 1^35^

*MiR* micro-ribonucleic acid.

### Myocardial ischaemia and injury

EVs and their miRs are key players in mediating inflammatory and coagulative reactions involving endotheliocytes, platelets, SMCs and inflammatory cells and thus contribute to the development of atherosclerosis and angiogenesis^11^. Endotheliocytes and platelets have been shown to be the major sources of EVs in patients with stable coronary artery disease (CAD). Atherosclerotic conditions promote the packaging of miR-92a-3p into endothelial EVs, which regulates angiogenesis in recipient endotheliocytes by a thrombospondin 1-dependent mechanism^12^. Coronary serum EVs isolated from patients with myocardial ischaemia promote angiogenesis via the miR-939-mediated nitric oxide signalling pathway^13^. EVs derived from activated endotheliocytes and inflammatory cells, such as monocytes, and especially those expressing cell adhesion and procoagulant molecules, indicate early vascular dysfunction^14^. Low mitochondrial encoded cytochrome c oxidase I (MT-COI) in monocyte-specific EVs from stable CAD patients is used to identify a population that is at risk for new cardiovascular events^15^. EVs from macrophage-derived foam cells can promote SMC migration and adhesion, which might be mediated by the integration of EVs into the SMCs and the subsequent activation of extracellular regulated protein kinase (ERK) and protein kinase B (PKB)^16^. Endothelial EV-mediated transfer of miR-126 promotes the repair of endotheliocyte damage caused by hyperglycaemic conditions. Intravascular injection of endothelial EVs containing miR-126 accelerated reendothelialization after electric denudation of the endothelium in vivo^17^. Moreover, endotheliocytes shed EVs containing miR-126 during atherosclerosis and induce the production of stromal cell-derived factor-1 (SDF-1) in recipient vascular cells. SDF-1 counteracts endotheliocyte apoptosis, recruits progenitor cells and improves plaque stability^18^. Increased expression of miR-126 and miR-199a in EVs but not plasma has been associated with reduced major adverse cardiovascular events in patients with stable CAD^19^.

Endotheliocytes with increased expression of intercellular adhesion molecule 1 (ICAM-1), an adhesion molecule, in the cell membrane show increased endothelial–monocyte adhesion in cell culture, and endothelial EVs play anti-inflammatory roles by reducing endothelial ICAM-1 expression through transferring functional miR-222 into recipient cells^20^. Hyperglycaemia-activated miR-503 is packaged into endothelial EVs and delivered to vascular pericytes, resulting in reduced expression of Ephrin-B2 (EFNB2) and vascular endothelial growth factor (VEGF) in these cells, followed by impaired migration and proliferation^21^. Shear stress and Kruppel-like factor 2 (KLF2), a shear-responsive transcription factor, cause miR-143 upregulation in endotheliocytes and control the phenotypes of SMCs. EVs derived from KLF2-expressing endotheliocytes reduce atherosclerotic lesion formation, improve atherosclerotic plaque stability and promote progenitor cell incorporation in the aorta in ApoE^−/−^ mice^22^. Mesenchymal stromal cell (MSC)-derived EVs attenuate myocardial ischaemia–reperfusion injury in mice by shuttling miR-182, which modifies the polarisation status of macrophages^23^. Hypoxia-induced MSC-derived EVs facilitate ischaemic cardiac repair through miR-125b-mediated amelioration of cardiomyocyte apoptosis and prevention of cardiomyocyte death in myocardial infarction (MI)^24^.

Cardiovascular risk factors change the content of plasma EVs, and EVs play a crucial role in intercellular signalling, influencing the development of myocardial injury^25^. Due to increased release from activated or apoptotic cells, EVs are increased in myocardial injury and represent biomarkers for MI^26^. In MI, cardiomyocytes secrete EVs enriched in miRs related to endothelial proliferation and differentiation, including miR-143 and miR-222, as a response to hypoxic conditions and inflammation^27^. A circulating EV protein network that is also involved in the inflammatory response has been found to be associated with MI^28^. Moreover, EVs play a fundamental role in the reorganisation of heart muscle after MI, suggesting an enormous potential for interventions by increasing or decreasing miR-containing EVs.

### Atrial enlargement and fibrillation

An enlarged left atrium is closely related to atrial fibrillation, and patients with atrial fibrillation develop cardiac tissue thickening or fibrosis. In patients with atrial fibrillation, miR-21-3p has been found in EVs and plasma and associated with abnormal enlargement of cardiac tissue. MiR-199a-3p in extracellular matrix-derived EVs has been shown to promote cardiac tissue growth and affect atrial electrical function by repressing homeodomain-only protein X (HOPX) expression and increasing Gata-binding 4 (Gata4) acetylation^29^.

### Cardiac hypertrophy and failure

EVs have been shown to mediate cross-talk between cardiomyocytes and fibroblasts in cardiac hypertrophy and have become disease biomarkers and diagnostic tools for cardiac hypertrophy and heart failure (HF). Previous studies have shown a significant role of miRs and proteins transferred by EVs in the development of HF and protection against undesired modifications to heart muscle. MiR-21-5p contributes to EV-mediated heart repair by enhancing angiogenesis and cardiomyocyte survival through the phosphatase and tensin homologue (PTEN)/PKB pathway. However, the pathological conditions of HF patients impair their regenerative activities by dysregulating miR-21-5p in cardiac-derived EVs^30^. Cardiac fibroblast-derived EVs enriched in miR-21-3p serve as paracrine signalling-mediators of cardiomyocyte hypertrophy. MiR-21-3p induces cardiomyocyte hypertrophy by targeting sorbin and SH3 domain containing protein 2 (SORBS2). Furthermore, pharmacological inhibition of miR-21-3p reduces the development of cardiac hypertrophy in an Ang II-induced mouse model^31^. EVs secreted from hypertrophied cardiomyocytes contain heat shock protein 90 (HSP90) and interleukin (IL-6) to maintain the signal transducer and activator of transcription 3 (STAT3) pathway signalling^32^. EVs released from diabetic cardiomyocytes inhibit the proliferation, migration, and tube formation of endotheliocytes through a mechanism involving the transfer of EVs containing miR-320^33^. MiR-146a-loaded EVs shed by endotheliocytes are messengers in the development of peripartum cardiomyopathy and biomarkers for the diagnosis of peripartum cardiomyopathy^34^. A 16 kDa N-terminal prolactin fragment (16 K PRL) induces increased miR-146a expression in endotheliocytes and promotes the release of miR-146a-loaded EVs. MiR-146a suppresses small ubiquitin-like protein modifier 1 (SUMO1) expression and induces cardiac dysfunction in maladaptive hypertrophy^35^.

### CVD prevention and treatment

MiR and protein contents have been shown to differ in the EVs from lean and fatty rats^36^. EVs released from hypertrophic adipocytes impair endotheliocyte function, suggesting a possible role for EVs in obesity-related atherosclerosis^37^. Adipocyte-derived EVs have been shown to promote macrophage activation by delivering adipocyte-dominant transcripts (mRNAs and miRs) to macrophages^38^. Adipocyte-derived EVs might inhibit the regulation of macrophage cholesterol efflux and promote the development of atherosclerosis^39^. Adipocyte-derived EVs promote plaque burden and exacerbate vulnerable atherosclerosis by inducing angiogenesis of the vasa vasorum in diabetic ApoE^−/−^mice^40^. MiR-93-5p-enhanced adipose-derived stromal cell-derived EVs prevent cardiac injury by inhibiting autophagy and inflammation and attenuate acute MI-induced myocardial damage^41^. Visceral fat-derived EVs, regardless of obesity, facilitate macrophage-derived foam cell formation through the prevention of adenosine triphosphate (ATP)-binding cassette transporter 1 (ABCA1)- and ATP-binding cassette subfamily G member 1 (ABCG1)-mediated cholesterol efflux^42^. Only obese visceral fat-derived EVs are capable of inducing macrophage polarisation. EVs from the abdominal adipose tissue of obese mice but not lean mice activate monocyte differentiation into macrophages and induce insulin resistance^43^. The lipid modifying enzyme phospholipase D2 (PLD2) has been demonstrated to be involved in EV production and acts downstream of the small guanosine triphosphatase (GTPase) adenosine diphosphate (ADP)-ribosylation factor 6 (ARF6). PLD2 might integrate a significant number of signalling pathways that participate in EV release. Long-term exercise-derived circulating EVs protect the heart from myocardial ischaemia/reperfusion injury via exosomal miR-342-5p^44^. Long-term exercise training potentially enhances silent information regulator 1 (SIRT1) signalling, attenuates cardiac inflammation, and provides cardioprotection in d-galactose-induced ageing rats^45^. Cigarette smoke-induced EVs promote thrombin generation in normal human plasma and contribute to increased cardiovascular risk in smokers^46^.

MiR and protein profiles have been shown to differ in EVs from hypertensive rats compared with normotensive rats^47^. Human embryonic kidney 293 cells containing SV40 large T antigen (HEK293T cells) overexpressing angiotensin II type 1 receptor (AT1R) secreted the EVs enriched in AT1Rs when subjected to osmotic stretch in vitro^48^. Likewise, in mice subjected to transverse aortic constriction to induce pressure overload, EVs were released into the serum that had a 100-fold increase in AT1R density. These AT1R-enriched EVs transferred functional AT1R to recipient wild-type HEK293T cells in vitro and to heart muscle in AT1R-knockout mice in vivo. There have been obvious increases in EVs in the plasma of diabetic individuals, and diabetic conditions render EVs ineffective in activating survival pathways in cardiomyocytes^49^. Insulin resistance increases EV secretion, and EVs might modulate insulin signalling because EVs are preferentially internalised by leucocytes and alter leucocyte function^50^. In the absence of diabetes mellitus, cardioprotective EVs have been shown to activate the toll-like receptor 4 (TLR-4)-extracellular signal-regulated kinase 1 and 2 (ERK1/2) pathway. Exosomal mammalian sterile 20-like kinase 1 (Mst1)-enriched EVs released from cardiac microvascular endothelial cells to cardiomyocytes worsen diabetic cardiomyopathy by inhibiting cardiomyocyte autophagy, promoting cardiomyocyte apoptosis and suppressing glucose metabolism^51^.

Additional investigations have explored the use of EVs as a delivery vehicle for various therapies because of their ability to easily cross biological membranes (Table 2)^52–54^. An engineered hydrogel patch capable of slowly releasing and providing sustained delivery of EVs secreted from induced pluripotent stem cell (iPSC)-derived cardiomyocytes has been developed to promote ejection fraction recovery, reduce arrhythmic burden, prevent cardiomyocyte apoptosis, reduce infarct size and inhibit cell hypertrophy when implanted onto rat hearts after MI^55^. In a preclinical MI mouse model, localised injection of miR-21-loaded EVs effectively delivered miR-21 into cardiomyocytes and endotheliocytes, drastically inhibited cell apoptosis and significantly restored cardiac function^56^. EVs engineered by ischaemic myocardium-targeting peptides can specifically target the ischaemic myocardium, and MSC-derived ischaemic myocardium-targeting peptide-containing EVs exert enhanced therapeutic effects on MI^57^.

**Table 2:** Extracellular vesicle-based therapeutic options in cardiovascular diseases.

Therapies	Roles
Intravascular injection of endothelial EVs containing miR-126^17^	Accelerating reendothelialization after electric denudation of the endothelium^17^
MSCs-derived EVs^23^	Modifying the polarisation status of macrophages and attenuating myocardial ischaemia–reperfusion injury^23^
Hypoxia-induced MSC-derived EVs^24^	Ameliorating cardiomyocyte apoptosis and preventing cardiomyocyte death in MI^24^
cTnI-targeted EVs carrying miR-590-3p from MSCs^70^	Promoting cardiomyocyte proliferation and restoring cardiac function in the peri-MI area^70^
An engineered hydrogel patch capable of slowly releasing and providing sustained delivery of EVs secreted from iPSC-derived cardiomyocytes^55^	Promoting ejection–fraction recovery, lowering arrhythmic burden, reducing infarct size and inhibiting cell hypertrophy after MI^55^
Cell-free delivery of EVs secreted from iPSC-derived cardiomyocytes^71^	Promoting heart recovery in myocardial injury^71^
Localised injection of miR-21-loaded EVs^56^	Inhibiting cell apoptosis and restoring cardiac function in preclinical MI^56^
EVs engineered by ischaemic myocardium-targeting peptides^57^	Enhancing therapeutic effects on MI^57^

*EV* extracellular vesicle, *MiR* micro-ribonucleic acid, *MSCs* mesenchymal stromal cells, *MI* myocardial infarction, *iPSCs* induced pluripotent stem cells.

### Stem cell-derived EVs

**Fig. 3:**
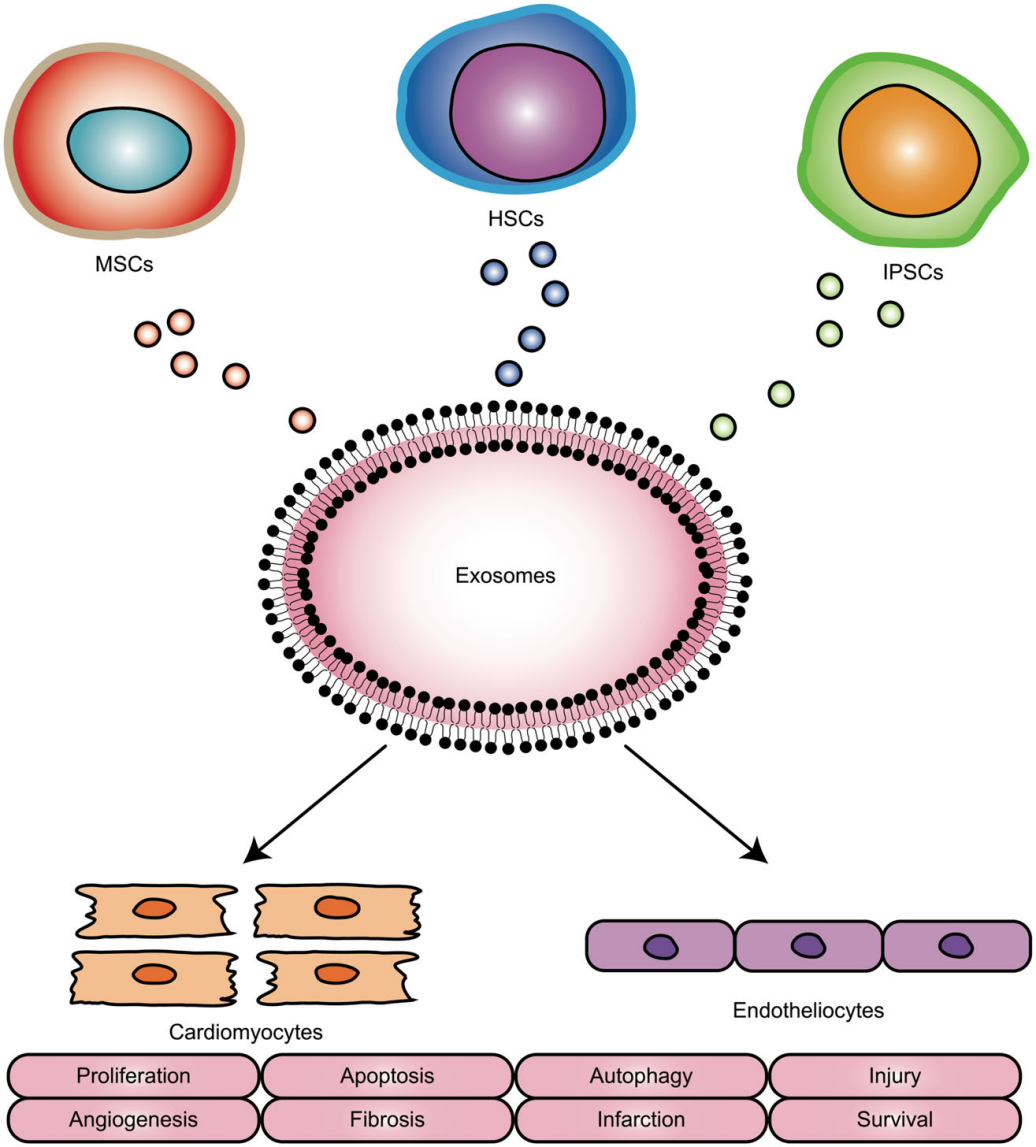
Origins and roles of stem cell-derived extracellular vesicles. Extracellular vesicles (EVs) mimic the cardioprotective properties of various stem cells, including mesenchymal stromal cells (MSCs), haematopoietic stem cells (HSCs) and induced pluripotent stem cell (iPSCs). These EVs modulate cardiomyocytes and endotheliocytes by stimulating cell proliferation, decreasing cell apoptosis, inhibiting cell autophagy, promoting angiogenesis, reducing tissue fibrosis, preventing cardiovascular injury, treating myocardial infarction and improving cardiac survival.

EVs mimic the cardioprotective properties of various stem cells (Fig. 3), including MSCs, haematopoietic stem cells (HSCs) and iPSCs^58–60^. These EVs modulate cardiomyocytes and endotheliocytes by stimulating cell proliferation, decreasing cell apoptosis, inhibiting cell autophagy, promoting angiogenesis, reducing tissue fibrosis, preventing cardiovascular injury, treating MI and improving cardiac survival. Several studies examining the roles of MSC-secreted EVs have found that these EVs are involved in cardioprotective paracrine effects^61^. MSCs originate in the bone marrow, and MSC-derived EVs influence both endotheliocytes and cardiomyocytes. MSC-derived EVs affect endotheliocytes and cardiomyocytes by stimulating angiogenesis and decreasing apoptosis by preserving mitochondrial membrane potential through miR-19a enrichment^62^. MSC-derived EVs can protect cardiac tissue from ischaemic injury by promoting blood vessel formation, reducing infarct size and preserving cardiac function^63^. MSCs have been shown to mediate cardioprotective paracrine effects by secreting EVs^64^. MSC–EVs significantly improve cardiac survival, enhance capillary density, reduce cardiac fibrosis and restore long-term function, suggesting that EVs released from MSCs, can act as shuttles and stimulate the proliferation and migration of cardiomyocytes^65^. Whereas EVs harvested from stem cells in vitro might protect against cardiac injury and promote cardiac repair, EVs secreted from endogenous cardiac cells might play a more deleterious role in the progression of CVDs. Circulating myocardial miRs from infarcted hearts, such as miR-1, miR-208 and miR-499, are carried in EVs and mobilise bone marrow progenitor cells^66^. Injured cardiomyocyte-derived EVs accelerate transplanted MSC injury in infarcted hearts^67^.

MiR-132, delivered by MSC-derived EVs, promotes angiogenesis in MI^68^. EVs derived from injured cardiomyocytes might impede the survival and promote the apoptosis of bone marrow-derived MSCs in the infarcted heart. Transplanted MSCs reduce autophagic flux in infarcted hearts via exosomal transfer of miR-125b^69^. cTnI-targeted EVs carrying miR-590-3p from MSCs were endocytosed by cardiomyocytes and thus promoted cardiomyocyte proliferation in the peri-MI area and eventually restored cardiac function as a treatment for MI^70^. Cell-free delivery of EVs secreted from iPSC-derived cardiomyocytes into the heart promotes heart recovery and treats myocardial injury^71^. EVs produced by cells under adverse conditions not only contribute to disease pathology but also might be detrimental towards cardiac regenerative therapies.

### Standardisation of EV studies

Scientific studies have sharply increased in the past 10 years, analysing multiple functions of EVs in numerous physiological pathways, from ageing, obesity, CVDs to other diseases. EV research has now clearly gained widespread interest and enthusiasm. However, the promotion of rigorous EV research is an ongoing process due to a lack of well-accepted standards for performing EV studies, which hinders progress in studying the role of EVs as diagnostic biomarkers and therapeutic options for different diseases. The conclusions of several EV studies are not sufficiently supported by the experiments performed or the information reported. The International Society for Extracellular Vesicles (ISEV) proposed the minimal information for studies of extracellular vesicles guideline in 2018. The major goal of this guideline was to guide experimental requirements specific to the EV field and promote standardisation in the EV field. By improving the reliability and reproducibility of EV studies, EVs will have stronger potential as diagnostic biomarkers and therapeutic options for CVDs and other diseases.

## Conclusion

Proper cardiovascular function depends on the coordinated interplay and communication between cardiomyocytes and noncardiomyocytes. Despite EVs representing a significant mechanism for intracellular communication, little is known about EV-mediated regulation of cardiomyocytes and noncardiomyocytes within the healthy and diseased heart. Moreover, how neighbouring or distant cells can secrete EVs to affect cardiac structure and function is largely unknown. On the one hand, miRs and proteins are transferred by EVs to maintain cardiovascular homeostasis, but on the other hand, the dysregulation of miRs and proteins transferred by EVs gives rise to the development of CVDs. Considering the fundamental roles of EVs in regulating physiological and pathological functions, future studies should be aimed at the broad and detailed understanding of EV functions and the development of new and personalised EV-based therapies.
